# Generation of qualified clinical-grade functional hepatocytes from human embryonic stem cells in chemically defined conditions

**DOI:** 10.1038/s41419-019-1967-5

**Published:** 2019-10-10

**Authors:** Zhongwen Li, Jun Wu, Lei Wang, Weifang Han, Juan Yu, Xin Liu, Yukai Wang, Ying Zhang, Guihai Feng, Wei Li, Glyn Nigel Stacey, Qi Gu, Baoyang Hu, Liu Wang, Qi Zhou, Jie Hao

**Affiliations:** 1 Savaid Medical School, University of Chinese Academy of Sciences, Beijing, 100049 China; 2 State Key Laboratory of Stem Cell and Reproductive Biology, Institute of Zoology, Chinese Academy of Sciences, Beijing, 100101 China; 3 Beijing Stem Cell Bank, Chinese Academy of Sciences, Beijing, 100190 China; 4 Institute for Stem Cell and Regeneration, Chinese Academy of Sciences, Beijing, 100101 China; 5 University of Chinese Academy of Sciences, Beijing, 100049 China; 6 International Stem Cell Banking Initiative, Hertfordshire, UK; 7 State Key Laboratory of Membrane Biology, Institute of Zoology, Chinese Academy of Sciences, Beijing, 100101 China

**Keywords:** Embryonic stem cells, Stem-cell differentiation

## Abstract

Hepatocytes have been successfully generated from human pluripotent stem cells (hPSCs). However, the cost-effective and clinical-grade generation of hepatocytes from hPSCs still need to be improved. In this study, we reported the production of functional hepatocytes from clinical-grade human embryonic stem cells (hESCs) under good manufacturing practice (GMP) requirements. We sequentially generated primitive streak (PS), definitive endoderm (DE), hepatoblasts and hepatocyte-like cells (HLCs) from hESCs in the different stages with completely defined reagents. During hepatoblast differentiation, dimethyl sulfoxide (DMSO), transferrin, L-ascorbic acid 2-phosphate sesquimagnesium salt hydrate (Vc-Mg), insulin, and sodium selenite were used instead of cytokines and FBS/KOSR. Then, hepatoblasts were differentiated into HLCs that had a typical hepatocyte morphology and possessed characteristics of mature hepatocytes, such as metabolic-related gene expression, albumin secretion, fat accumulation, glycogen storage, and inducible cytochrome P450 activity in vitro. HLCs integrated into the livers of Tet-uPA Rag2^–/–^ Il2rg^–/–^ (URG) mice, which partially recovered after transplantation. Furthermore, a series of biosafety-related experiments were performed to ensure future clinical applications. In conclusion, we developed a chemically defined system to generate qualified clinical-grade HLCs from hESCs under GMP conditions. HLCs have been proven to be safe and effective for treating liver failure. This efficient platform could facilitate the treatment of liver diseases using hESC-derived HLCs transplantation.

## Introduction

As one of the most important organs in the body, the liver is mainly responsible for metabolism, detoxification, protein synthesis, and production of biochemicals necessary for digestion^1^. Furthermore, the liver is susceptible to many major diseases, such as liver fibrosis, cirrhosis, hepatitis, and liver cancer, which are the main causes of morbidity and mortality^2,3^. Patients with acute liver injuries or end-stage liver diseases have no choice but to receive a liver transplant. The liver is an unique in the organ because it has the capability to regenerate an intact liver with normal physiological functions even if only 25% of the liver remains^4^. However, the source of donor livers is restricted, which leads to mortality among patients while they wait for a liver transplant. In recent years, the development of liver cell transplantation has provided an alternative strategy for the treatment of liver diseases. Hepatocyte transplantation has been attempted to treat liver failure clinically^5–10^. It has been reported that patients with acquired liver diseases, such as acute liver failure, fulminant hepatic failure and chronic liver diseases, benefited from the hepatocyte infusion either with or without liver transplantation. However, the use of primary or fetal hepatocytes has been restricted due to the lack of available healthy donors as well as limited cell proliferation, functional deficits, the risk of immune rejection, and the concern regarding ethical issues^11,12^. Therefore, other types of hepatocytes, hepatocyte-like cells (HLCs), which can be derived from human pluripotent stem cells (hPSCs), including human embryonic stem cells (hESCs) and induced pluripotent stem cells (iPSCs), have been reported to improve liver function after transplantation into animal models with liver injuries^13–20^. However, there are also some concerns regarding the regeneration potentials of HLCs compared with primary or fetal hepatocytes. Accordingly, no clinical trials with HLCs have been conducted because of the concerns regarding whether HLCs have a comparable function to primary human hepatocytes and the questionable biosafety for HLCs^21,22^.

Several studies have reported methods for differentiating hESCs into hepatocyte-like cells^13,15,17,18,23,24^. Deng et al. developed an effective three-stage method using serum-free medium^17^. Subsequently, rigorous protocols were established using cytokines, such as FGF2, BMP4, HGF, and KGF^25,26^, or cost-effective small molecules with FBS/KOSR^27,28^.

These studies make have indicated the possibility for clinical application of hepatocytes-like cells derived from hPSCs. However, to the best of our knowledge, in spite of significant effort by numerous groups, there are still no successfully generated qualified defined hepatocytes from hPSCs and previously reported studies lack biosafety-related experiments, which has hindered clinical application.

Recently, several attempts on the generation of GMP-grade HLCs have been reported. Hay and colleagues (2017) developed a process to generate hepatocyte-like cells, which was reproducible, cost-effective and can decrease batch-to-batch variation^29^. Rashid et al. (2019) reported the generation of hepatocyte-like cells using clinical-grade hPSCs under 3D culture conditions^30^. These studies have indicated the possibility for clinical application of hepatocytes-like cells derived from hPSCs. However, to the best of our knowledge, in spite of significant effort by numerous groups, there are still no successfully generated qualified defined hepatocytes from hPSCs and previously reported studies lack biosafety-related experiments, which has hindered clinical application.

In this study, we report the generation of qualified clinical-grade functional hepatocytes derived from a clinical-grade hESC cell line (Q-CTS-hESC-2) that has been generated specifically for use in human therapy and biobank in GMP conditions^31^. We generated HLCs by sequentially directing hESCs through the primitive streak (PS), definitive endoderm (DE) and hepatoblast lineages, thus, mimicking the natural development process. These HLCs possessed the typical hepatocyte morphology and characteristics of mature hepatocytes, such as metabolic-related gene expression, albumin secretion, fat accumulation, glycogen storage, and inducible cytochrome P450 activity in vitro. Furthermore, upon transplantation into URG mice, liver failure was induced under uPA activation, and the hESCs-derived HLCs were able to integrate into the host liver and restore liver function. Moreover, a series of biosafety-related experiments were performed to ensure future clinical applications.

## Materials and methods

All reagents used for clinical hESC culture and hepatocyte-like cell differentiation are shown in Supporting Table S1.

### Ethics statement

All procedures in this study were completed under the guidelines of the Institute of Zoology, Chinese Academy of Sciences and were approved by the Institute of Zoology, Chinese Academy of Sciences.

### Cell culture

The clinical hESC line (Q-CTS-hESC-2) was prepared as described previously^31^. Clinical hESCs were maintained in commercially available Essential 8^TM^ basal medium (E8) (Life Technologies) on vitronectin-coated plates (1 µg/cm^2^). Cells were passaged every 5 or 6 days using Versene (Life Technologies). Clinical hESCs were tested weekly for mycoplasma contamination using a Myco-detection Kit (InvivoGen) and endotoxin contamination was tested using a ToxinSensor^TM^ Chromogenic LAL Endotoxin Assay Kit (GenScript, L00351). All cultures were maintained at 37 °C, 5% CO_2_ and atmospheric O_2_ in a humidified incubator (Thermo Fisher Scientific). The frozen primary human hepatocytes (PHHs) were purchased from the Research Institute for Liver Diseases (Shanghai, China) and were maintained at 37 °C and 5% CO_2_ in HI Medium (HIM) (Research Institute for Liver Diseases) prior to being used for experiments.

### Hepatic cell differentiation

Clinical-grade hESCs were digested into single cells using CTS-Tryple (Life Technologies). The cells were collected by centrifugation at 1200 rpm for 3 min and then resuspended in E8 medium containing 10 µM ROCK inhibitor (Y-27632). The cell suspension was seeded to Matrigel (Corning)-coated plates at a concentration of 2 × 10^4^ viable cells per cm^2^. After 24 h incubation, hESCs were cultured in RPMI-1640 medium (Life Technologies) supplemented with 100 ng/mL Activin A (R&D Technologies) and 25, 50, and 100 ng/mL Wnt3a (R&D Technologies) or 2, 3, and 5 µM GSK3 inhibitor CHIR99021 (Stemgent) for 1 day. Then, the medium was changed to RPMI-1640 medium supplemented with 1 × CTS-B27 (Life Technologies) and 100 ng/mL Activin A for 2 days. On day 3, the medium was changed to CTS-KO-DMEM/F12 (Life Technologies) supplemented with 1 × non-essential amino acids (NEAA) (Life Technologies), 1 × CTS-GlutaMAX^TM^ (Life Technologies), 5 mg/mL transferrin (Sigma-Aldrich), 50 µg/L L-ascorbic acid 2-phosphate sesquimagnesium salt hydrate (Vc-Mg) (Sigma-Aldrich), 10 µg/mL insulin (Sigma-Aldrich), 0.1 ng/mL sodium selenite (Sigma-Aldrich), and 1% DMSO (Sigma-Aldrich). After 6 days, the medium was changed to Iscove’s-modified Dulbecco’s medium (IMDM) (Life Technologies) supplemented with 20 ng/mL HGF, 20 ng/mL oncostatin M (OSM) (R&D Technologies), 10 µM dexamethasone (Dex) (Sigma-Aldrich), 2 µM SB431542 (Stemgent), and 1 µM RO4929097 (Selleck) for 10 days.

### PCR

For reverse transcription polymerase chain reaction (RT-PCR) analysis, RNA was extracted using an RNAprep Pure Cell/Bacteria Kit (TIANGEN, DP430). For PHHs, mRNA was extracted from PHHs cultured for 24 h after plating. Two micrograms of RNA was reverse transcribed into cDNA using a PrimeScript™ First-Strand cDNA Synthesis Kit (TaKaRa, 6110B). Real-time quantitative PCR (qPCR) was performed and analyzed using a Stratagene Mx3005P system (Agilent Technologies) with SYBR Green Real-Time PCR Master Mix Plus (Toyobo, QPS-201). A GAPDH transcript was used for internal normalization.

### Immunofluorescence staining

Cells were fixed with 4% paraformaldehyde (PFA) (ALADDIN Chemical Co., Ltd., C104190) for 15 min, permeabilized with PBS containing 0.5% Triton X-100 for 15 min and blocked with PBS containing 2% bovine serum albumin (BSA) for 60 min at room temperature. Then, the cells were stained with 1:200 goat polyclonal IgG FOXA2 (R&D Technologies, AF2400), 1:200 goat polyclonal IgG SOX17 (R&D Technologies, AF1924), 1:200 mouse monoclonal IgG HNF4α (Abcam, ab41898), 1:200 mouse monoclonal IgG AFP (Sigma-Aldrich, A8452), 1:200 mouse monoclonal IgG ALB (R&D Technologies, MAB1455), 1:200 mouse monoclonal IgG CK18 (Abcam, ab2254), 1:250 mouse monoclonal IgG AAT (Sigma-Aldrich, SAB4200198), and 1:200 mouse monoclonal IgG ASGPR1 (Thermo Fisher Scientific, MA1-40244) overnight at 4 °C in 2% BSA in PBS. Then, the cells were washed three times with PBS and then incubated with 1:200 Alexa Fluor 488 donkey anti-mouse IgG (H + L) (Jackson ImmunoResearch, 705-546-147) or 1:200 Fluorescein (FITC) donkey anti-goat IgG (H + L) (Jackson ImmunoResearch, 715-096-150) secondary antibodies in 2% BSA in PBS for 60 min at room temperature in the dark. The washing step was repeated before staining the nuclei with Hoechst 33342 (Invitrogen, H3570).

### Flow cytometry

Cells were harvested and fixed with 4% PFA for 10 min, permeabilized and blocked with PBS containing 0.5% Triton X-100 and 2% BSA for 60 min at room temperature. Then, the cells were stained with primary antibodies overnight at 4 °C in 2% BSA in PBS. Then, the cells were incubated with secondary antibodies for 1 h in the dark at room temperature. For flow cytometry analyses of ASGPR1, Tra-1-81, Tra-1-60, and SSEA4, cells were only incubated with conjugated antibodies for 1 h at room temperature after fixation. After incubation, cells were washed twice and analyzed with a MoFlo (Beckman) and associated software. The antibodies used for flow cytometry were as follows: 1:200 goat polyclonal IgG FOXA2 (R&D Technologies, AF2400), 1:200 goat polyclonal IgG SOX17 (R&D Technologies, AF1924), 2 µg/10^6^ cells of mouse monoclonal IgG HNF4α (Abcam, ab41898), 1:200 mouse monoclonal IgG AFP (Sigma-Aldrich, A8452), 0.25 µg/10^6^ cells of mouse monoclonal IgG ALB (R&D Technologies, MAB1455), 10 µL/10^6^ cells of rabbit anti- human ASGPR1-FITC (Miltenyi Biotec, 130-109-490), mouse anti-SSEA4-PE (BD Pharmingen, 560128), mouse Alexa Fluor 488-conjugated anti-Tra-1-60 (Millipore, MAB4360A4), mouse Alexa Fluor 488-conjugated anti-TrA-1-81 (Millipore, MAB4381A4), Alexa Fluor 488 donkey anti-mouse IgG (H + L) (Jackson ImmunoResearch, 705-546-147), and fluorescein (FITC)-conjugated donkey anti-goat IgG (H + L) (Jackson ImmunoResearch, 715-096-150).

### Human albumin ELISA

To determine the secretion of human albumin, cell culture supernatants were collected after 48 h of culture. For PHHs maintained in HIM, supernatants were also collected after 48 h of culture. For transplantation experiments, animal blood was harvested through the tail vein and placed at room temperature for 10 min. Then, serum was collected by centrifugation at 5000 rpm for 15 min. The levels of human albumin were measured by a Human Albumin ELISA Kit (Assaypro, EA3201-1) according to the manufacturer’s instructions.

### Assays for PAS, ac-LDL, ICG, and oil red O staining

Cells were stained by periodic acid-Schiff (PAS) (Sigma-Aldrich, 395B) and DiI-ac-LDL (Biomedical Technologies, BT-902) following the manufacturer’s instructions. For the indocyanine green (ICG) (Sigma-Aldrich, I2633) uptake assay, the cells were changed to medium with 1 mg/mL ICG and incubated at 37 °C for 1 h. The cells were washed three times with PBS and imaged using an inverted microscope (Leica). For Oil red O staining, cells were washed three times with PBS and fixed with 4% PFA for 30 min. The ells were washed three times with PBS and then stained with Oil red O (Sigma-Aldrich, 00625) dissolved in isopropanol for 30 min. Then, the cells were washed twice with 60% isopropanol and imaged by inverted microscopy.

### CYP induction and metabolic assay

For the measurement of CYP enzyme induction, HLCs and PHHs were cultured with drugs. In detail, cells were incubated for 48 h in the presence of specific inducers, including 25 µM b-naphthoflavone (Sigma-Aldrich, N3633) for cytochrome P450 family 1 subfamily A polypeptides 1 and 2 (*CYP1A1* and *1A2*), 25 µM rifampicin (Sigma-Aldrich, R3501) for *CYP2A6*, *2C8*, and *3A4*, or an equal volume of DMSO as a vehicle control with replenishment every 24 h. Different CYP450 subunit induction expression levels were measured by qPCR. Different CYP450 activities were measured using commercially available cell-based assays (P450-Glo™ Assays; Promega Corporation, Madison, WI, USA).

### The genome variant calling

The 30X whole-genome sequencing data which produced by HiSeq X-Ten (Annoroad Gene Technology Co., Ltd) were used to analyze mutation changes between HLCs and ESCs. As previously described^32^, the whole-genome sequencing data were mapped to hg19 genome by BWA (version 0.7.15) using ‘mem’ mode with default parameters. The mapped reads with unique genome location were used for identify the single nucleotide variants (SNVs) and insertions and deletions (InDels) events by MuTect2 tool in GATK^33^. Only no <10 reads covered sites in both samples and the variants with 2 or more reads supported were kept. Then the variants located in repeats and low-complexity regions annotated by RepeatMasker (db20140131) were removed. According to the previous publication, the homopolymer region in genome also likely led to sequencing errors^32^. We also filtered the sites located within long homopolymer regions (≥5 bp).

### RNA-seq library preparation and data analysis

Total RNA was isolated from HLCs and PHHs by Trizol (Invitrogen). RNA-seq libraries were prepared with the NEBNext®Ultra^TM^ RNA Library Prep Kit for Illumina®. Sequencing was performed on an Illumina HiSeq X-Ten sequencer with 150 bp paired-end sequencing reaction. The bulk RNA-Seq data for hESCs were downloadwd from GEO database^34^. All RNA-seq data analysis was performed with Hisat2 (version 2.1.0)^35^ and Cufflinks (version 2.2.1)^36^ using the UCSC hg19 annotation with default settings. Reads with unique genome location and genes with no <1 FPKM in at least one sample were used for next analysis (Gene expression in each sample was shown in Supplementary Table S4). Fourfold change was used as the threshold to filter the differentially expressed genes. The Gene Ontology analysis for differentially expressed genes was performed by DAVID (version 6.8)^37^. Clustering and heatmap analysis were performed with function hierarchical cluster (person method) and heatmap.2 function in R.

### Transplantation of HLCs into URG mice

Male Tet-uPA Rag2^–/–^ Il2rg^–/–^ (URG) mice (8 weeks old) with a BALB/c background were purchased from Beijing Vitalstar Biotechnology (Beijing, China). URG mice were maintained in specific pathogen-free (SPF) conditions. The Institutional Animal Care and Use Committee of Institute of Zoology, Chinese Academy of Sciences approved all animal procedures. Twelve hours before cell transplantation, URG mice were intraperitoneally injected with 15 mg/kg body weight doxorubicin (Dox) (Sigma-Aldrich, D1515). Dox (0.25 mg/mL) was also administered through drinking water in the first week after cell transplantation. One week after cell transplantation, the concentration of Dox in drinking water for URG mice was increased to 0.5 mg/mL for the following 6 weeks. HLCs (*n* = 5) and PHHs (*n* = 5) (2 × 10^6^ cells/animal) in 50 µL PBS or 50 µL PBS (control, *n* = 5) were intrasplenically transplanted into URG mice. Blood samples were collected weekly by tail vein, and human albumin was quantified using a Human Albumin ELISA Kit (Assaypro, EA3201-1).

### Histology and immunohistochemistry

Tissues were fixed overnight with 4% paraformaldehyde and embedded in paraffin. Paraffin sections (5 µm thickness) were used for immunohistochemistry (IHC). For IHC, tissue sections were stained with monoclonal mouse anti-human hepatocyte-specific antigen CK8/18 (clone 5D3; Mai Xin, China), and immunoreactivity was detected with an Ultrasensitive^TM^ Streptavidin-Peroxidase Kit (KIT-9710, Mai Xin, China) according to the manufacturer’s protocol. The area of positively stained cells per field was assessed using ImageJ (NIH), and the percentage of HLC cell integration was analyzed.

### Tumorigenicity

Cells were harvested and suspended in CTS-DPBS at a density of 5 × 10^7^ cells/mL. Then, 20 µL of cell suspension was carefully injected into each testis of 6- to 8-week-old SCID mice (Beijing Vitalstar Biotechnology) using a sterile glass needle under a sterile stereo microscope. Two months post injection, the mice were euthanized, and the teratomas were examined following the guidelines of the Institutional Animal Care and Use Committee.

### Biosafety evaluation

The tested items are listed in Table S2. The “Pharmacopoeia of the People’s Republic of China, Edition 2015, Volume III”, was used as a reference for the testing methods.

### Statistics

Data are presented as the mean ± SD. Analysis of variance (ANOVA) was used to determine significant differences with *p* = 0.05 for statistical significance. Statistical calculations were performed using the Statistical Program for Social Sciences software (SPSS, IBM).

## Results

### Generation of qualified clinical-grade hepatocyte-like cells

**Fig. 1:**
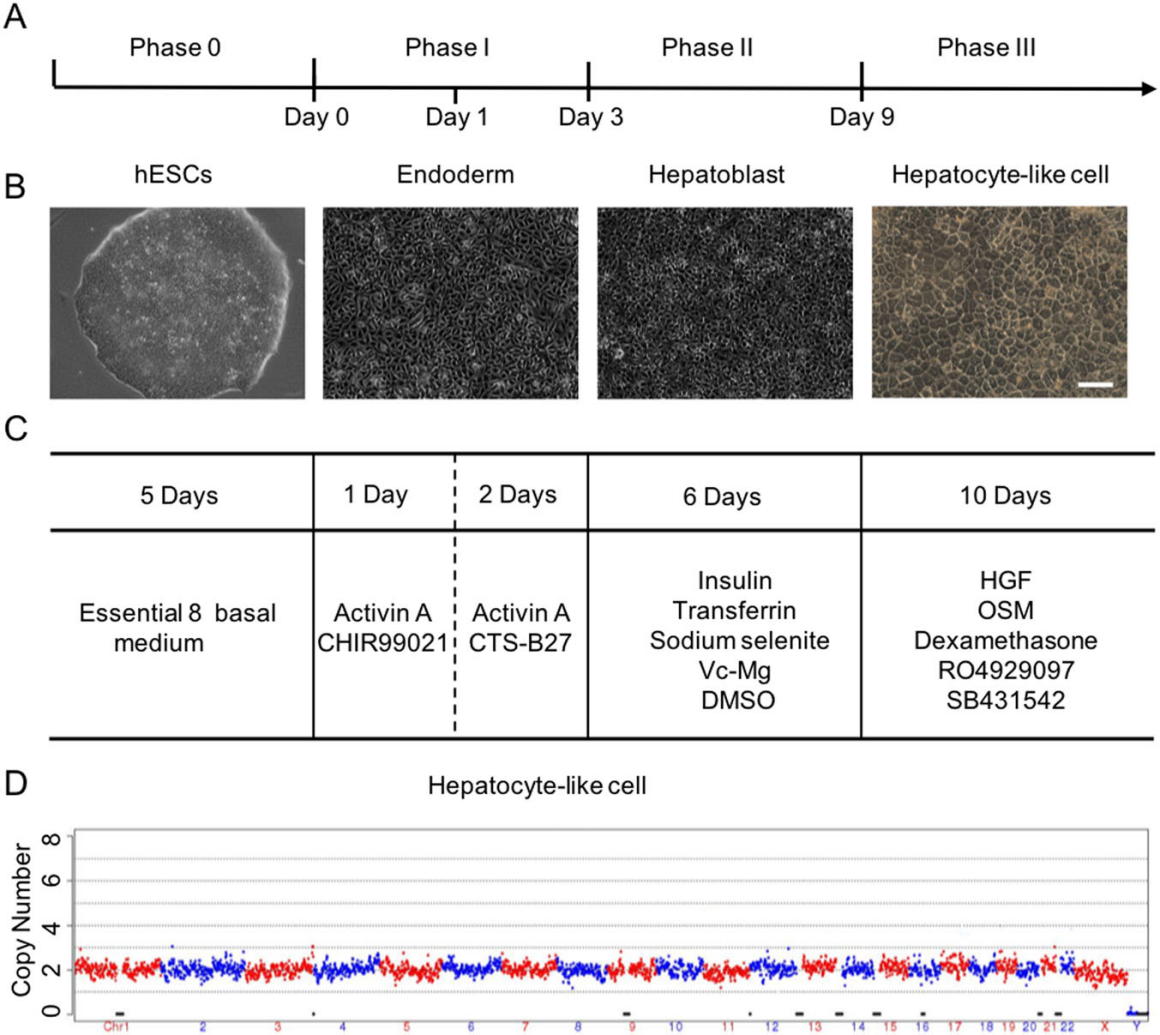
Overview of the differentiation of hepatocyte-like cells (HLCs) from human embryonic stem cells (hESCs). **a** The normal process of differentiation and the phases of the protocol used in this study. **b** Representative morphology of cells at different stages observed by phase contrast microscopy. **c** Summary of the time course and the additions of small molecules for each phase of differentiation. **d** Copy-number variation (CNV) analysis of hepatocytes from human embryonic stem cells. Scale bars, 200 µm.

In this study, we sequentially generated primitive streak (PS), definitive endoderm (DE) and finally hepatoblasts and hepatocyte-like cells (HLCs) from clinical-grade hESCs in different stages with completely xeno-free reagents (Table S1). Our differentiation strategy is illustrated in Fig. 1. HLCs derived from hESCs possessed the typical morphology of liver cells showing polygonal in shape and obvious nucleus (Fig. 1b). Moreover, copy-number variation sequencing (CNV-seq) further indicated that there was no chromosome aneuploidy and no loss or repeat >10 Mbp segments in HLCs (Fig. 1d). The somatic mutation analysis by whole-genome sequencing also showed less mutation level in differentiating from hESCs to HLCs compared with somatic cell passaging^38^ (Fig. S1a, b). Flow cytometry data showed no residual hESCs were detected on day 19 (Fig. S2a). Accordingly, there was no teratoma formation observed after HLCs injection into the testes of NOD-SCID mice (Fig. S2b, c). In addition, the system has been proven effective for other human embryonic stem cell lines (Fig. S3). To ensure safety, a series of biosafety-related experiments were performed according to the “Guidelines for Human Somatic Cell Therapies and Quality Control of Cell-based Products” and the HLCs were verified as suitable for use in human therapy (Table S2). The HLCs were qualified by the National Institutes for Food and Drug Control (NIFDC) in China.

### Differentiation of hESCs into definitive endoderm cells using Activin A and CHIR99021 instead of Wnt3a

**Fig. 2:**
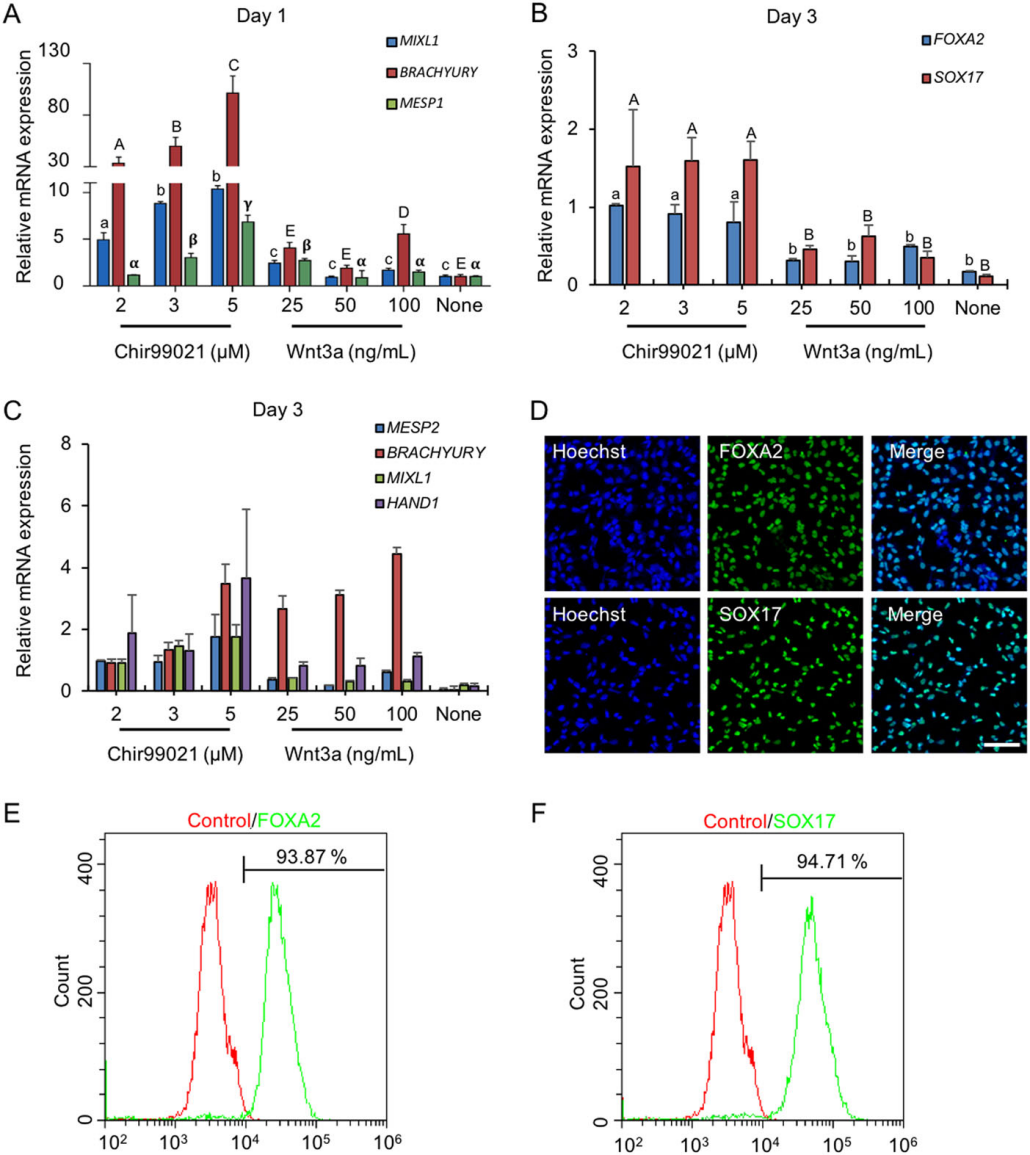
Differentiation of hESCs into definitive endoderm cells by Activin A with CHIR99021. **a** The relative primitive streak (*BRACHYRUY*, *MESP1*, and *MIXL1*) gene expression of the day 1 differentiated cells by adding Activin A (100 ng/mL) with CHIR99021 (2–5 µM) or Wnt3a (25–100 ng/mL) treatments were determined by real-time quantitative PCR (qPCR). None, no WNT signaling pathway activators were used on day 1 for PS differentiation (Activin A only). **b**, **c** After 1 day, the medium was changed to Activin A (100 ng/mL) and 1 × CTS-B27 to induce DE differentiation. The definitive endoderm (*FOXA2* and *SOX17*) and mesoderm (*MESP2* and *HAND1*) relative gene expression levels were determined by qPCR. **d** Immunofluorescence analysis of the expression of SOX17 and FoxA2 for Activin A with 2 µM CHIR99021-induced differentiated cells on day 3. **e**, **f** The expression of SOX17 and FoxA2 for Activin A with 2 µM CHIR99021-induced differentiated cells was determined by flow cytometry on day 3. Isotype control antibodies were used as controls. At a specific gene expression, datum points lacking common letters differ, *p* < 0.05. Data are represented as the mean ± SD. Scale bar, 100 µm.

Definitive endoderm (DE) differentiation has been achieved by using a series of growth factors and small molecules through mimicking in vivo liver development^17,25–28^. However, DE cells from these protocols were usually composed of mixed cell populations, which led to a low efficiency of endodermal derivatives^39^. DE cells arise from the primitive streak (PS, E6.5) in vivo^40,41^. Addition of TGFβ and WNT was founded to be critical on day 1 for hESCs to differentiate into PS cells^42^. Wnt3a has been reported to promote DE generation by activating WNT signaling^20,43^. Considering the high cost and instability of the Wnt3a protein, a phosphatidylinositol 3-kinase (PI3K) inhibitor, CHIR99021, was used to induce PS differentiation in this study. We found that the PS could be induced using 2–5 µM CHIR99021 (Fig. 2a) on day 1. Gene expression analysis showed that the CHIR99021-treated group was associated with higher transcript levels for PS compared with the Wnt3a-treated group by RT-qPCR. Furthermore, PS formation also failed using Activin A alone without activating WNT signal (Fig. 2a). Thus, even though Activin A was necessary, Activin A alone was insufficient to generate the PS. Therefore, we concluded that exogenous WNT agonists, either Wnt3a or CHIR99021, are required for PS induction. However, WNT signaling activation was initially essential for PS induction within the first 24 h and then repressed DE formation from the PS in the next stage from days 2 to 3^42^. Therefore, Wnt3a or CHIR99021 was removed after 24 h by changing medium supplemented with Activin A and CTS-B27 to induce DE differentiation. After 2 days of treatment, the expression of DE marker genes, such as *SOX17* and *FOXA2*, was determined (Fig. 2b). The results demonstrated that CHIR99021 induced higher expression levels of DE markers compared with those in Wnt3a-treated cells on day 1. Similarly, we observed that DE formation failed when WNT signaling was inactivated on day 1. On day 3, we also detected the expression of PS markers (*BRACHYRUY*, *MESP1*, and *MIXL1*) and mesoderm markers (*MESP2* and *HAND1*) (Fig. 2c). Two micromoles of CHIR99021 on day 1 was the most suitable concentration for DE formation with lower PS and mesoderm marker expression levels on day 3, which resulted in more than 90% of the differentiated cells expressing definitive endoderm marker genes, such as SOX17 and FoxA2 (Fig. 2d–f). These results indicated that the combination of Activin A and CHIR99021 could induce efficient DE formation in this study.

### Hepatoblasts differentiated from DE cells in defined xeno-free conditions

**Fig. 3:**
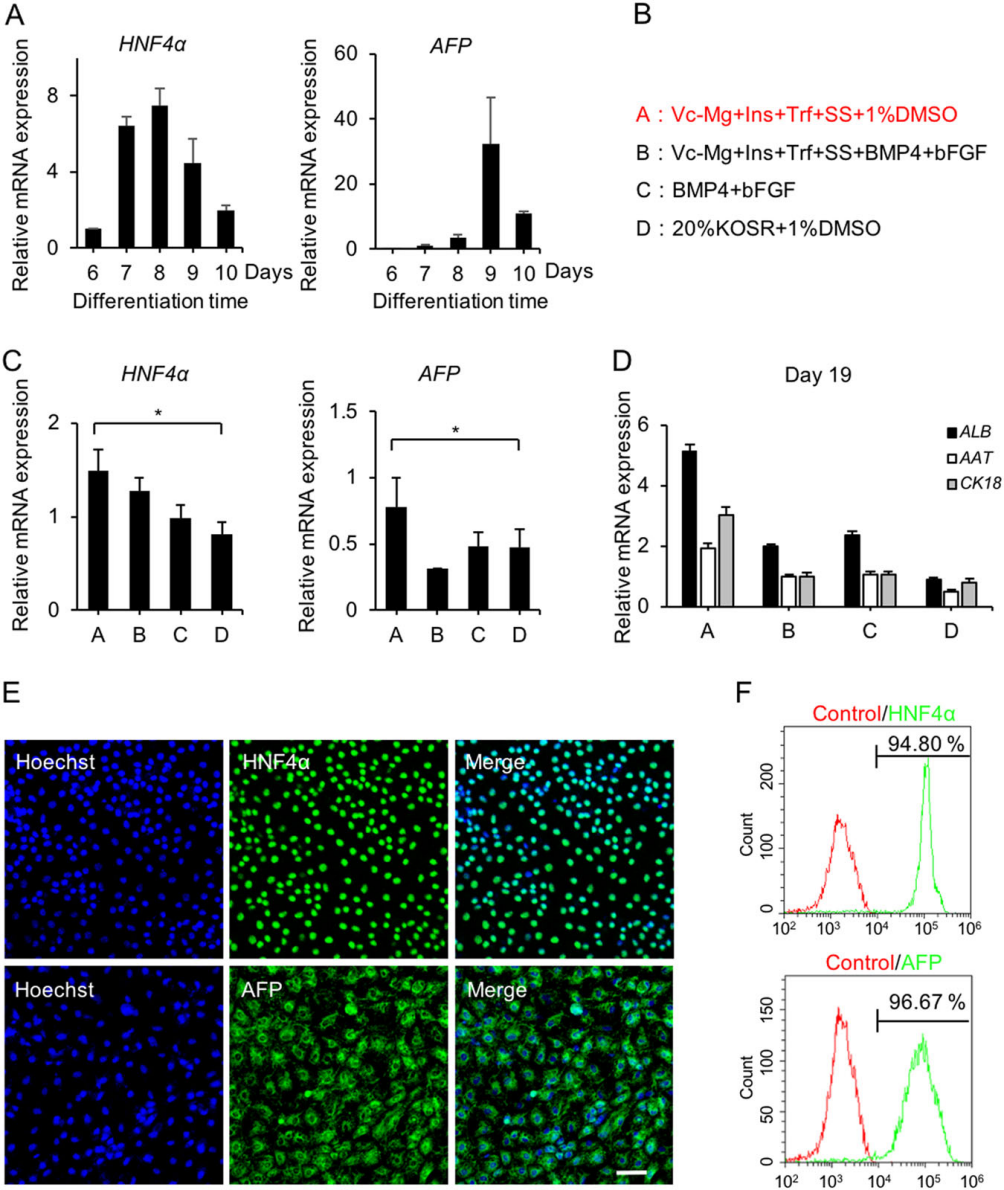
Differentiation of hESCs into hepatoblasts in defined xeno-free conditions. **a** The relative hepatoblast gene (*HNF4α* and *AFP*) expression levels of cells differentiated with a previously reported protocol were determined by qPCR at days 6, 7, 8, 9, and 10. **b** Four protocols were used to induce PE to hepatoblasts. Trf (transferrin, 5 mg/mL); Vc-Mg (L-ascorbic acid 2-phosphate sesquimagnesium salt hydrate, 50 µg/L); Ins (insulin, 10 µg/mL); SS (sodium selenite, 0.1 ng/mL); and BMP4 (10 ng/mL); bFGF (10 ng/mL). **c** The relative hepatoblast gene (*HNF4α* and *AFP*) expression of day 9 differentiated cells in basal medium (DMEM/F12, 1 × L-GlutaMAX, and 1 × NEAA) with different treatments (groups A, B, C, and D) were determined by qPCR. **d** The relative hepatocyte gene (*ALB*, *AAT*, and *CK18*) expression of day 19 differentiated cells in basal medium (DMEM/F12, 1 × L-GlutaMAX, and 1 × NEAA) with different treatments (groups A, B, C, and D) were determined by qPCR. **e** Immunofluorescence analysis of HNF4α and AFP expression in group A-induced differentiated cells on day 9. **f** The expression levels of HNF4α and AFP in group A-induced differentiated cells were determined by flow cytometry on day 9. Isotype control antibodies were used as controls. *p < 0.05, **p < 0.01; data are represented as the mean ± SD. Scale bar, 100 µm.

It has been reported that in mouse liver development, fibroblast growth factor (FGF) signaling from cardiac mesoderm and bone morphogenetic protein (BMP) signaling is essential for hepatic specification from the foregut endoderm in mouse liver development^44–46^. FBS or KOSR was also necessary for hepatoblast differentiation^13–16^. However, FGF and BMP as well as FBS or KOSR, are relatively expensive or originate from animals. We established a defined xeno-free differentiation system containing four factors (transferrin, Vc-Mg, insulin, and sodium selenite) and DMSO upon the substitution of FGF, BMP, and FBS or KOSR. The expression profile of *HNF4α* and *AFP* confirmed significantly increased expression levels of hepatoblast-related genes on day 9 post induction using a previously reported protocol^20^ (Fig. 3a). Then, we tried to induce hepatoblasts from DE cells using four different methods (Fig. 3b), in which Group A demonstrated the highest efficiency for inducing hepatoblast marker expression of *HNF4α* and *AFP* on day 9 (Fig. 3c) that was corroborated by immunophenotyping, which revealed more than 90% of the differentiated cells expressing hepatoblast markers HNF4α and AFP (Fig. 3e, f). We next investigated hepatocyte maturation of differentiated hepatoblasts into HLCs according to a previously reported protocol^20^, which demonstrated higher expression levels of hepatocyte-specific markers in Group A compared with those in other treatments (Fig. 3d). These findings affirmed the efficacy of our described xeno-free system for differentiating hPSCs into hepatoblasts.

### HLCs induction antagonized cholangiocyte

**Fig. 4:**
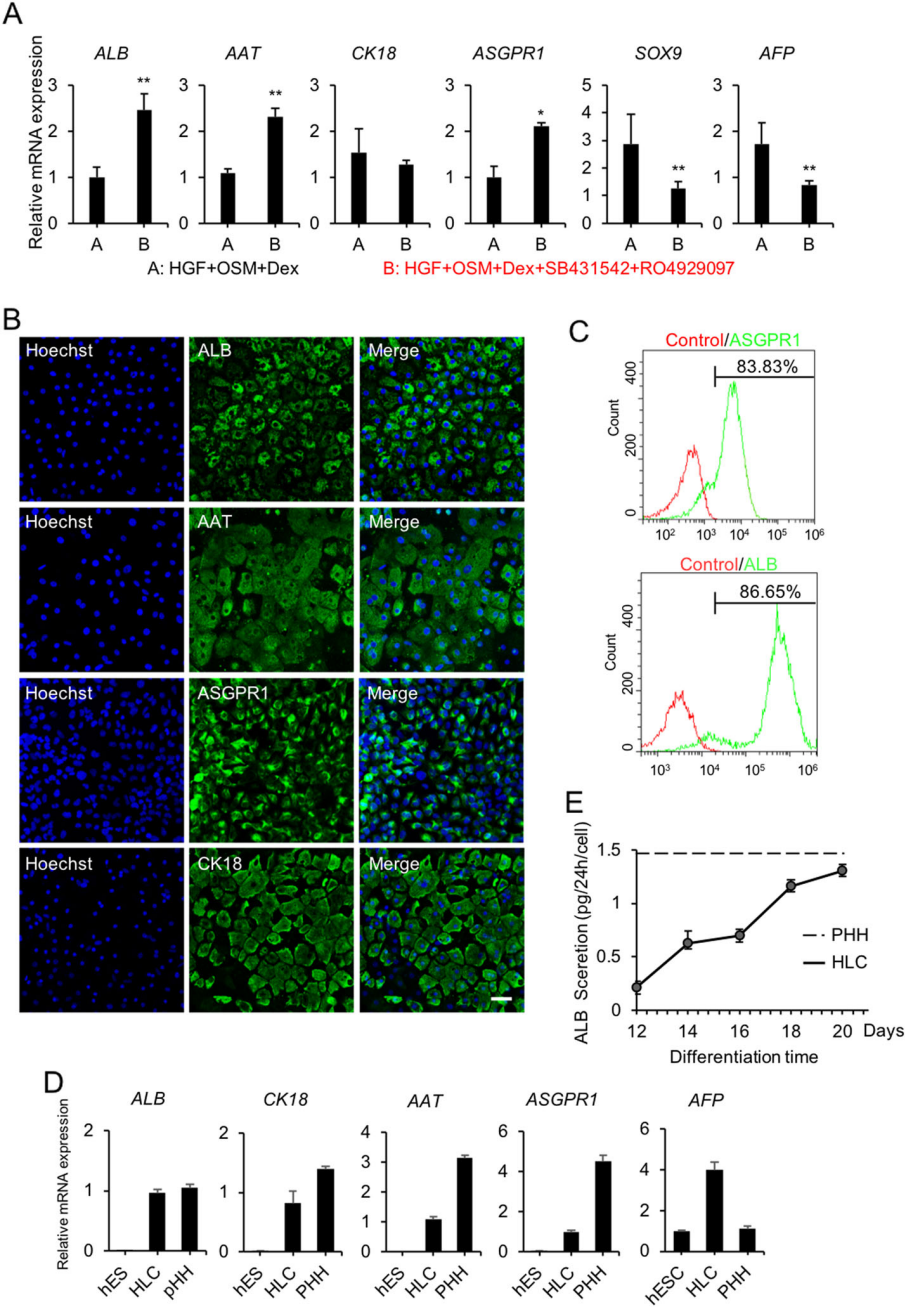
Differentiation of hESCs into HLCs. **a** The relative hepatocyte (*ALB*, *AAT*, *CK18*, and *ASGPR1*), cholangiocyte (*SOX9*), and hepatoblast (*AFP*) gene expression levels of day 19 differentiated cells with different treatments (groups A and B) were determined by qPCR. HGF (hepatocyte growth factor, 20 ng/mL); OSM (oncostatin M, 20 ng/mL); Dex (dexamethasone, 10 µM); SB431542 (TGFβ inhibitor, 2 µM); and RO4929097 (Notch inhibitor, 1 µM). **b** Immunofluorescence analysis of ALB, AAT, ASGPR1, and CK18 expression in group B-induced differentiated cells on day 19. **c** The expression levels of ALB and ASGPR1 in group B-induced differentiated cells were determined by flow cytometry on day 19. Isotype control antibodies were used as controls. **d** The relative hepatocyte (*ALB*, *AAT*, *CK18*, *ASGPR1*, and *AFP*) gene expression levels of differentiated HLCs (group B) compared with those of hESCs and primary human hepatocytes (PHHs) were determined by qPCR. **e** Albumin secretion of HLCs (black line) on days 12, 14, 16, 18, and 20 and PHHs (dotted line) on day 2 were determined by ELISA. *p < 0.05, **p < 0.01; data are represented as the mean ± SD. Scale bar, 50 µm.

Hepatoblasts have bidirectional differentiation potentials to either hepatocytes and cholangiocytes^47–49^. HGF, OSM, and Dex have been reported as usually being used in the induction of HLCs from hepatoblasts, while the method of differentiating hepatoblasts into cholangiocytes has not been fully addressed, which eventually leads to a mixed product of HLCs and cholangiocytes^13–20^. TGFβ, Notch, WNT, BMP, or FGF signaling regulates the differentiation of hepatoblasts into hepatocytes or biliary cells ^50–52^. It has been reported that inhibition of TGFβ and Notch signaling prevents biliary differentiation in vivo^51,53^. In this study, we inhibited the differentiation of cholangiocytes from hepatoblasts by the addition of 2 µM SB431542 (TGFβ inhibitor) and 1 µM RO4929097 (Notch inhibitor) in Group B (Fig. 4a). The results showed that the higher expression levels of hepatocyte-specific markers and the lower expression levels of cholangiocyte marker *SOX9* and immature marker *AFP* were observed in Group B compared with those in Group A processed according to the previously reported protocol (Fig. 4a). Immunofluorescence staining demonstrated that HLCs expressed the hepatocyte markers ALB, AAT, ASGPR1, and CK18 (Fig. 4b). Furthermore, the flow cytometry results showed that more than 80% of the differentiated cells expressed hepatocyte-specific proteins ASGPR1 and ALB (Fig. 4c). Although the mRNA levels of hepatocyte-specific markers were lower (higher for AFP) in HLCs than primary human hepatocytes (PHHs), comparable levels of plasma protein ALB secretion were determined in HLCs according to the results of the ELISA assay (Fig. 4d, e).

### HLCs possessing CYP enzyme activity, biliary excretion capability and hepatocyte-specific function

**Fig. 5:**
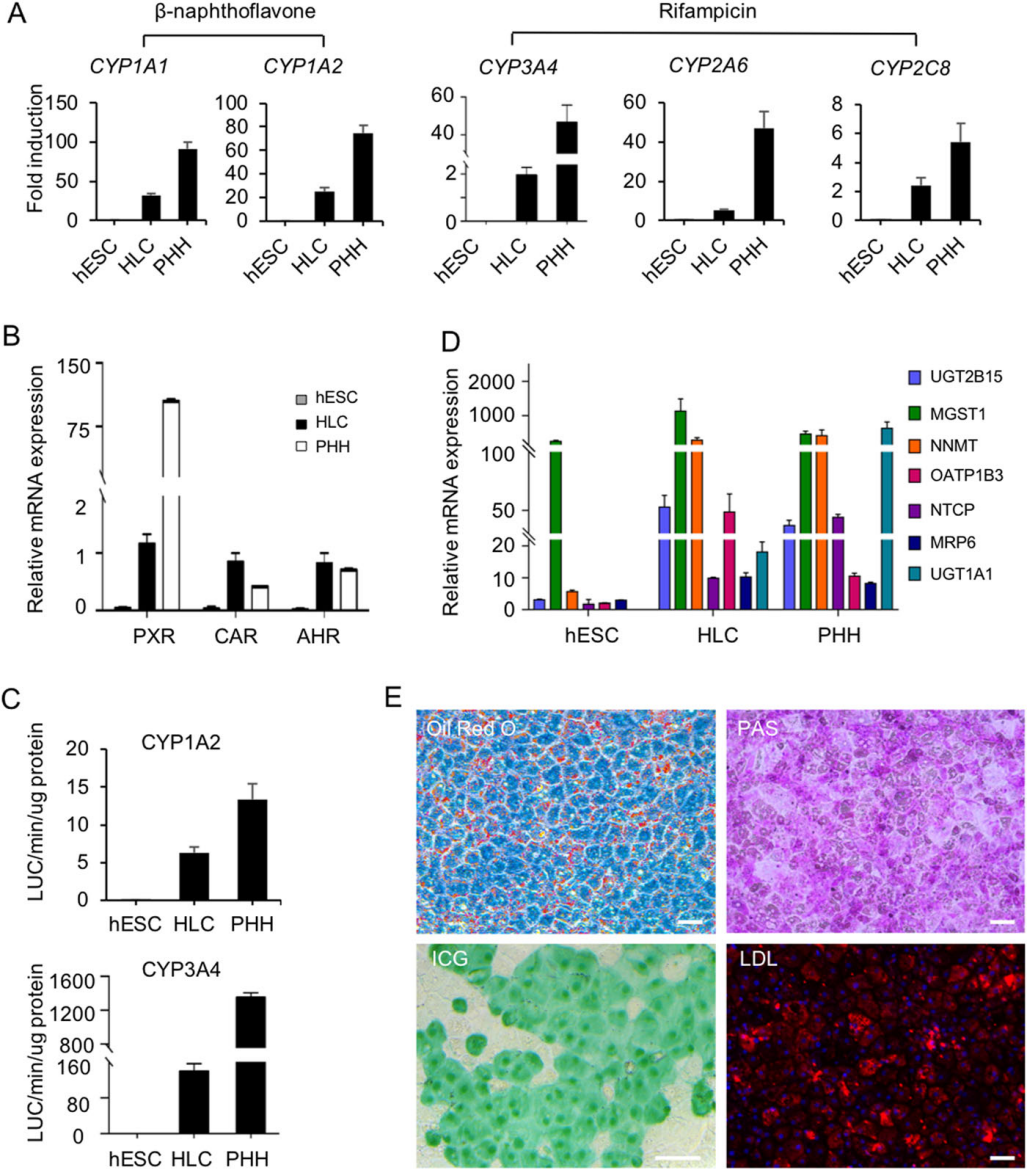
HLCs possessed CYP enzyme activities, biliary excretion capability, and hepatocyte-specific functions. **a** Expression levels of the induced CYP enzymes were measured by qPCR. *CYP1A1*, and *CYP1A2* were induced by 25 µM β-naphthoflavone. *CYP3A4*, *CYP2A6*, and *CYP2C8* were induced by 25 µM rifampicin. Fold-induction in HLCs and PHH cells were normalized to the levels in cells without induction treatment. **b** The mRNA levels of detoxification-related nuclear receptors were measured by qPCR in HLCs and PHHs cultured for 2 days. **c** CYP3A4 and CYP1A1 activities were measured with Luciferin-IPA and Luciferin-CEE, respectively. **d** Expression levels of drug transporter genes in HLCs were determined by qPCR. **e** HLCs showed comparable adipogenesis (Oil red O staining), glycogen accumulation (PAS staining), ICG intake and DiI-ac-LDL intake. Data are represented as the mean ± SD. Scale bar, 50 µm.

To further determine the functions of HLCs in vitro, we examined whether HLCs could respond to the treatments of CYP enzyme inducers. CYP450 enzymes were expressed at certain levels in normal culture medium without additional chemical induction. It has been reported that CYP450 gene transcription is usually regulated by the nuclear receptors AHR (CYP1A1 and CYP1A2), CAR (CYP3A4 and CYP2C8), and PXR (CYP2A6, CYP2C8, and CYP3A4) and these nuclear receptors are highly expressed in HLCs (Fig. 5b). After 48 h of *β*-naphthoflavone or rifampicin induction, mRNA expression levels of CYP genes, *CYP1A1* and *1A2*, or *CYP2A6*, *CYP2C8*, and *CYP3A4*, were significantly upregulated, and subsequently, the enzymatic activities of CYP3A4 and CYP1A1, the most important metabolic CYPs in the liver, were confirmed using luciferase assays (Fig. 5a, c). The results of qPCR also indicated high expression levels of some key transporter genes in HLCs (Fig. 5d). Furthermore, HLCs had mature hepatocyte-specific functions including glycogen storage, acetylated low density lipoprotein (ac-LDL) intake, indocyanine green (ICG) absorption and cytoplasmic accumulation of neutral triglycerides and lipids (Fig. 5e).

**Fig. 6:**
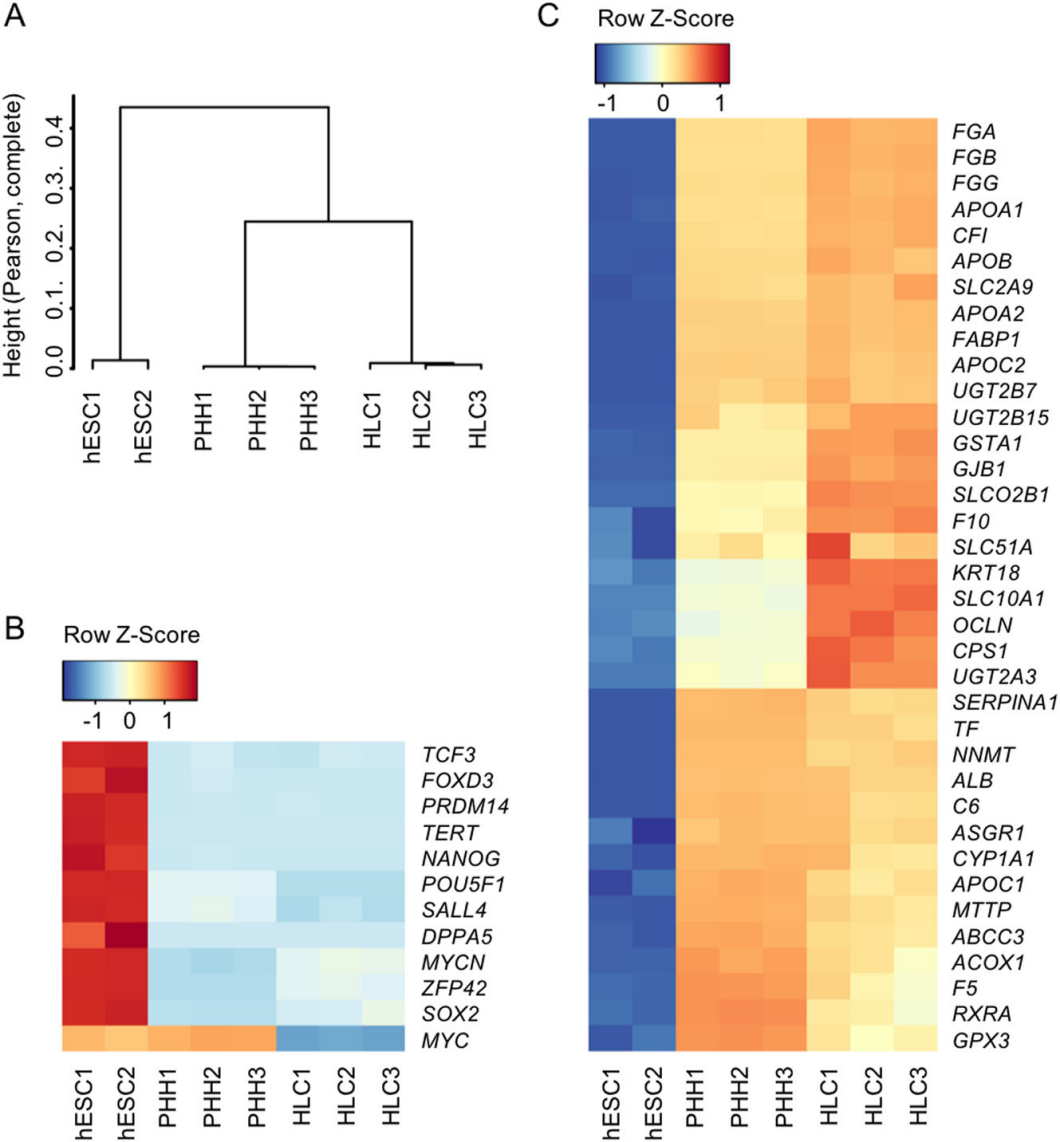
Transcriptome analysis of HLCs. **a** Unsupervised hierarchical clustering analysis based on the gene expression levels of each cell type. **b** Heatmap showing pluripotency marker gene expression changes. **c** Heatmap showing functional hepatocyte gene expression changes.

Further, we performed genome-wide profiling of HLCs and PHHs and compared their gene expression with hESCs^34^. Whole-genome analysis using principal component analysis (PCA) confirmed that HLCs clustered together with PHHs in an unsupervised hierarchical clustering analysis, suggesting similarity of their global expression profiles (Fig. 6a). Accordingly, pluripotency genes were significantly extinguished in HLCs and PHHs (Fig. 6b). The global differential expressed gene analysis showed that highly expressed genes in HLCs and PHHs compared with ESCs, were enriched with lipid metabolism related processes (Fig. S4). And the results are consistent with the liver related metabolism function of HLCs. Next, we analyzed the expression profile of hepatocyte-specific genes (Fig. 6c). Similar to PHHs, HLCs showed completely different gene expression patterns compared with hESCs. Interestingly, we found that some genes showed higher expression levels in HLCs than PHHs. These genes involved fat digestion and absorption (*APOB*, *APOA1*, *FABP1*, and *APOC2*), complement and coagulation cascades (*FGG*, *FGB*, *CFI*, and *F10*), phase II metabolic enzymes (*UGT2A3*, *UGT2B15*, and *UGT2B7*), tight junctions (*OCLN* and *GJB1*) and bile secretion (*SCL51A*, *SLCO2B1*, and *SLC10A1*). These results may provide insight regarding the use of HLCs. In summary, these data indicated that HLCs possessed characteristics of mature liver cells in vitro.

### Repopulation of tet-uPA Rag2^–/–^ Il2rg^–/–^ mouse livers with HLCs

**Fig. 7:**
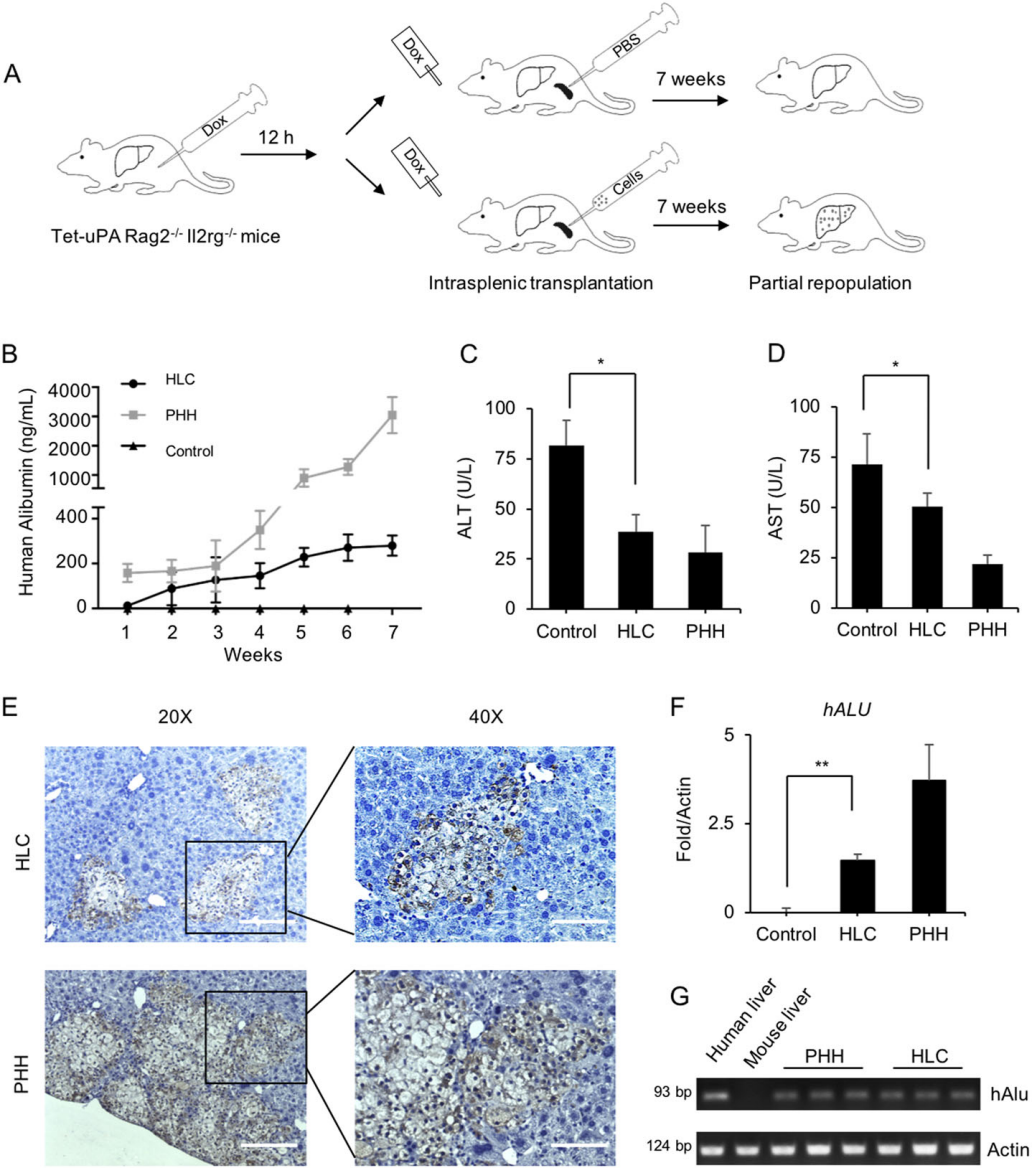
Repopulation of tet-uPA Rag2^–/–^ Il2rg^–/–^ mouse livers with HLCs. **a** Schematic outline of HLC transplantation into the livers of Tet-uPA Rag2^–/–^ Il2rg^–/–^ mice. Doxycycline (Dox) was injected into mouse abdomens 12 h before cell transplantation. Dox was administered through drinking water after cell transplantation. HLCs (2 × 10^6^ cells, *n* = 5), PHHs (2 × 10^6^ cells, *n* = 5) or PBS (*n* = 5) as the control were intrasplenically transplanted into mice. Animals were monitored daily and sacrificed 7 weeks after transplantation. **b** Human albumin levels in mouse serum were monitored weekly by ELISA. **c**, **d** Serum levels of ALT and AST were determined at 7 weeks following transplantation in mouse serum among the control (*n* = 4), HLC (*n* = 5), and PHH (*n* = 5) groups. **e** The integration of HLCs and PHH cells in URG mouse livers were determined by immunostaining for human CK18 in serial sections. **f**, **g** Expression levels of the human hepatocyte-specific gene *ALU* in the liver tissues from HLC- and PHH-transplanted URG mice (HLC, *n* = 5; PHH, *n* = 5) and control mice (*n* = 4). The values were normalized to total (mouse plus human) levels of *ACTIN*. *p < 0.05. Data are represented as the mean ± SD. For ×20 magnification, the scale bar represents 200 µm; and for ×40 magnification, the scale bar represents 100 µm.

Next, we investigated the regenerative capability of HLCs by injecting the cells intrasplenically into Tet-uPA (urokinase-type plasminogen activator) Rag2^–/–^/Il2rg^–/–^ mice with drug-induced liver failure under uPA activation with Dox administration through injection and drinking water^54^ (Fig. 7a). To eliminate the interference of mouse serum albumin, the specificity of the human albumin ELISA kit was tested. Results showed that the kit had no cross-reactivity with mouse albumin (Fig. S5a). After HLC transplantation, human albumin secretion in mouse serum gradually increased, and at week 7, it reached the maximum level of 321 ± 44 ng/mL, which was much lower than 3.45 ± 0.62 µg/mL human albumin secretion that was detected in PHH-treated animals (Fig. 7b). Importantly, the concentrations of ALT and AST in mouse serum were significantly decreased to comparable levels in both HLCs and PHHs (Fig. 7c, d). Immunohistochemical staining of human CK18 revealed that HLCs restored ~3 ± 1.7% of the recipient liver (Fig. 7e and Fig. S5b, c). Real-time PCR of the human-specific *ALU* sequence further confirmed the colonization of HLCs in recipient livers (Fig. 7f, g). Accordingly, human-specific gene *ALB*, human hepatocyte-specific metabolic gene phase I enzymes *CYP1A2* and *CYP2A6*, phase II enzyme *UGT1A1*, and human-specific transporters *MRP2* and *NTCP* were detected in recipient livers (Fig. S5d). No tumorigenesis was observed in transplant recipients at week 7 after either HLC or PHH injection. Overall, these data suggested that HLCs could integrate into URG mouse livers and ameliorate liver dysfunction caused by uPA accumulation.

## Discussion

Recently, hepatocytes derived from hPSCs have attracted much attention worldwide due to their vast potential for liver regeneration. This study aimed to develop and characterize a system that allows efficient generation of clinical-grade HLCs. DE fate determination is the key step enabling HLC generation from hESCs. In vivo, TGFβ and WNT signaling orchestrate DE differentiation from the PS. Nevertheless, Wnt3a plays an important role in PS induction^55^, and we conducted a feasibility assessment of Wnt3a replacement with other commercially available low-cost WNT agonists, such as CHIR99021, because of the high cost and instability of Wnt3a. CHIR99021 treatment resulted in a higher efficiency of PS induction compared with Wnt3a in the first 24 h. Nevertheless, the persistent activation of BMP and WNT signaling potently inhibited DE formation in the following stage. Loh et al. reported that either BMP or WNT signaling inhibition is sufficient to abolish mesoderm formation; however, this was not observed in this study^42^. Therefore, we subsequently removed only CHIR99021 when inducing the DE lineage. RPMI-1640 supplemented with B27 is the most commonly used basal medium for DE differentiation. To address the issues associated with xenogeneic contamination from conventional B27, a xeno-free CTS-B27 supplement was used to induce the DE in this study. Concerns regarding high cost and variability of cell factors and other components described were overcome^27,28^ by using a defined chemical formulation (containing transferrin, Vc-Mg, insulin, and sodium selenite) that enabled DE formation without adding FBS or KOSR.

To account for the bidirectional differentiation potential of hepatoblasts to either hepatocytes or cholangiocytes, high-purity HLCs should be generated by suppressing cholangiocyte formation. In this study, TGFβ and Notch inhibitors were introduced to prevent the induction of cholangiocytes from hepatoblasts, and high-purity HLCs were observed to express high levels of hepatocyte-specific markers, including *ALB*, *AAT*, *CK18*, and *ASGPR1*. Moreover, HLCs possessed CYP enzyme activities and displayed numerous functions of mature hepatocytes. However, the HLCs were not equivalent to adult hepatocytes. The mRNA level of *AFP* was higher in HLCs than in PHHs. This may indicate that the differentiation state of the HLCs was still at an early stage. In future, differentiation time of the protocol will be extended. In addition, many 3D differentiation systems have been reported to generate mature hepatocytes that better mimic the in vivo situation^24,56,57^. Hence, a 3D culture system can be used to improve the differentiation of hESCs toward mature hepatocytes. Moreover, the percentage of ALB-positive cells was 86%. Further work is planned to understand the nature of the remaining population before HLCs can be used in human therapy.

To investigate the capacity of HLCs to repopulate mouse livers, URG mice were injected intrasplenically with HLCs. Interestingly, the data showed that the secretion of albumin was only 3 µg/mL after PHHs transplantation. However, Deng et al. reported that the secretion of albumin was 150 µg/mL after transplanting PHHs into URG mouse livers^58^. The source variation of primary hepatocytes between donations may be one reason for this disparity, because frozen PHHs were used in this study. Importantly, HLCs repopulated 1.1–4.7% of the liver parenchyma in the recipients. The repopulation rate was low and the human albumin level was also not robust in the mice at 7 weeks. It would be important to enhance albumin level by extending experiments to around 12~16 weeks. In addition, serum levels of ALT and AST were significantly reduced in the HLCs recipients. Therefore, our generated HLCs could potential enable recovery of liver failure. In the future, further animal studies in different disease models will be necessary to verify the effectiveness of HLCs.

In recent years, HLCs have been generated from hESCs through various protocols. The generation of clinical-grade HLCs is not dependent on one guideline but rather multiple requirements, which must be met. First, the generation of clinical-grade hESCs was strictly performed as described previously under GMP conditions^31^. After HLCs were generated with a chemically defined system, a series of biosafety-related experiments were performed according to NIFDC guidelines. These biosafety-related experiments included testing for bacteria, fungi, mycoplasma, virus (by in vivo and in vitro method), pluripotent cell residuals, tumorigenicity testing, and biopreparation testing (endotoxin assay and bovine serum albumin residuals). These data indicated that HLCs safety in this study is in accordance with the efficacy validation results for future applications in liver therapeutics.

In conclusion, we developed a safety-qualified system to generate clinical-grade HLCs from hESCs under GMP conditions. HLCs have been proven to be safe and effective for treating liver failure and our results provide a platform for the development of HLC-based cell therapies.

## Supplementary information

Supplemental Information

Supplementary Table S4
