# Correction: Generation of qualified clinical-grade functional hepatocytes from human embryonic stem cells in chemically defined conditions

**DOI:** 10.1038/s41419-020-2259-9

**Published:** 2020-01-23

**Authors:** Zhongwen Li, Jun Wu, Lei Wang, Weifang Han, Juan Yu, Xin Liu, Yukai Wang, Ying Zhang, Guihai Feng, Wei Li, Glyn Nigel Stacey, Qi Gu, Baoyang Hu, Liu Wang, Qi Zhou, Jie Hao

**Affiliations:** 1 Savaid Medical School, University of Chinese Academy of Sciences, Beijing, 100049 China; 2 State Key Laboratory of Stem Cell and Reproductive Biology, Institute of Zoology, Chinese Academy of Sciences, Beijing, 100101 China; 3 Beijing Stem Cell Bank, Chinese Academy of Sciences, Beijing, 100190 China; 4 Institute for Stem Cell and Regeneration, Chinese Academy of Sciences, Beijing, 100101 China; 5 University of Chinese Academy of Sciences, Beijing, 100049 China; 6 International Stem Cell Banking Initiative, Hertfordshire, UK; 7 State Key Laboratory of Membrane Biology, Institute of Zoology, Chinese Academy of Sciences, Beijing, 100101 China

**Keywords:** Embryonic stem cells, Stem-cell differentiation

**Correction to**: **Cell Death and Disease**

10.1038/s41419-019-1967-5 published online 10 October 2019

The original version of this article contained an error in the name of one of the co-authors (Glyn Nigel Stacey). This has been corrected in the PDF and HTML versions of the article.

