# Supplementary material for: Generation of qualified clinical-grade functional hepatocytes from human embryonic stem cells in chemically defined conditions
Source: Cell Death Dis. 2019 Oct 10;10(10):763. doi: 10.1038/s41419-019-1967-5 (PMC6787193; doi:10.1038/s41419-019-1967-5)
Supplement: Supplementary file 1 — Supplemental Information [file 41419_2019_1967_MOESM1_ESM.docx]

**Generation of qualified clinical-grade functional hepatocytes from** **human embryonic stem cells in chemically defined conditions**

**Supplementary Table S1 GMP reagents for hESCs culture and hepatocyte-like cells differentiation.**

| **Reagent** | **Supplier** | **Catalog Number** | **Regulation** |
| --- | --- | --- | --- |
| KnockOut™ SR Xeno Free CTS™ (CTS-KOSR) | Life Technologies | 12618012 | Manufactured under cGMP with DMF |
| CTS™ TrypLE™ Select Enzyme (CTS-Tryple) | Life Technologies | A12859-01 | Manufactured under cGMP with DMF |
| CTS™DPBS (CTS-DPBS) | Life Technologies | A1285801 | Manufactured under cGMP with DMF |
| Essential 8™ Medium (E8) | Life Technologies | A1517001 | Manufactured under cGMP |
| Vitronectin | Life Technologies | A14700 | Manufactured under cGMP |
| Versene | Life Technologies | 15040066 | Manufactured under cGMP |
| Non Essential Amino Acid (NEAA) | Life Technologies | 11140050 | Manufactured under cGMP |
| CTS™ GlutaMAX™-I Supplement (CTS-GlutaMAX) | Life Technologies | A12860-01 | Manufactured under cGMP with DMF |
| CTS™ KnockOut™ DMEM/F-12 (CTS-KO-DMEM/F12) | Life Technologies | A13708 | Manufactured under cGMP with DMF |
| CTS™ B-27® Supplement (CTS-B27) | Life Technologies | A14867 | Manufactured under cGMP with DMF |
| Y-27632 | Selleck | S1049 | Manufactured under cGMP |
| CHIR99021 | Stemgent | 04-0004 | Manufactured under cGMP |
| Matrigel | Corning | 354277 | Manufactured under cGMP |
| Transferrin | Sigma-Aldrish | T3309 | ≤1.0 EU/mg endotoxin, Manufactured under cGMP |
| Sodium Selenite | Sigma-Aldrish | 71950 | PREMIUM Level, Manufactured under cGMP |
| L-ascorbic acid 2-phosphate Sesquimagnesium Salt Hydrate (Vc-Mg) | Sigma-Aldrish | A8960 | Manufactured under cGMP |
| RO4929097 | Selleck | S1575 | Manufactured under cGMP |
| SB431542 | Stemgent | 04-0010 | Manufactured under cGMP |
| RPMI-1640 | Life Technologies | 31800-022 | Manufactured under cGMP with Type II DMF |
| Dimethyl Sulfoxide (DMSO) | Sigma-Aldrish | D2438 | GMP Level, BioPerformance Certified |
| IMDM | Life Technologies | 12440053 | Manufactured under cGMP and ISO 13485 standard |
| Oncostatin M (OSM) | R&D | 295-OM/CF | Manufactured under cGMP |
| HGF | R&D | 294-HG/CF | Manufactured under cGMP |
| Dexamethasone (Dex) | Sigma-Aldrish | D4902 | Manufactured under cGMP |
| Insulin | Sigma-Aldrish | 91077c | GMP Level, Manufactured under cGMP |
| Activin A | R&D | 338-AC/CF | Manufactured under cGMP |
| Wnt 3a | R&D | 5036-WN-010/CF | Manufactured under cGMP |

**Supplementary Table S2 Biological safety analysis of the qualified clinical-grade hepatocyte-like cells.**

| **Sterility and pathogen**^a^ | **Hepatocyte-like cells** |
| --- | --- |
| **[Identification tests]** |  |
| Cell morphology | Adherent cells in monolayer, nucleus is apparent, visible polygon shape, showing hepatocyte-like morphology |
| Isozyme analysis | B type of human origin |
| Short tandem repeats (STRs) | Expressing 16 STR loci, each STR locus has 1-2 alleles. STR data is consistent with its original ESCs. |
| **[Bacteria and fungi]** | Negative |
| **[Mycoplasma]** | Negative |
| **[Exogenous virus test - in vitro]** |  |
| Cell observation | Negative |
| Hemadsorption test | Negative |
| Hemagglutination test | Negative |
| **[Exogenous virus test - in vivo]** |  |
| Cell inoculation in suckling mice | Survival rate 100% |
| Cell inoculation in adult mice | Survival rate 100% |
| Cell inoculation in guinea pigs | Survive, no tuberculosis |
| Cell inoculation in rabbits | Survive, no abnormality |
| Survival rate of 5- to 6-day-old chick embryos | Survival rate >90% |
| Hemagglutination test of 9- to 11-day-old chick embryo allantoic fluid | Negative |
| **[Biological effectiveness test]** |  |
| SOX17 expression assay by immunofluorescence | Positive, while its original ESCs is negative |
| HNF4a expression assay by immunofluorescence | Positive, while its original ESCs is negative |
| ALB expression assay by immunofluorescence | Positive, while its original ESCs is negative |
| HNF4a expression assay by PCR | Positive, while its original ESCs is negative |
| ALB expression assay by PCR | Positive, while its original ESCs is negative |
| Human albumin ELISA | Positive, while its original ESCs is negative |
| Oil red O staining | Positive, while its original ESCs is negative |
| DiI-ac-LDL absorb tests | Positive, while its original ESCs is negative |
| **[****Pluripotent cells residuals]** |  |
| TRA-1-60+ proportion assay by FACS | < 0.1% |
| SSEA-4+ proportion assay by FACS | < 0.1% |
| TRA-1-60+ proportion assay by FACS | < 0.1% |
| OCT4 expression assay by immunofluorescence | Negative, while its original ESCs is positive |
| NANOG expression assay by immunofluorescence | Negative, while its original ESCs is positive |
| OCT4 expression assay by PCR | Negative, while its original ESCs is positive |
| Nanog expression assay by PCR | Negative, while its original ESCs is positive |
| Teratoma formation in SCID mice | Six weeks after cell inoculation in SCID mice, no teratoma formation was observed, while its original ESCs is positive |
| **[Tumorigenicity test]** |  |
| Nude mice inoculated | Negative |
| Soft agar clone formation assay | Negative |
| **[Biopreparate test]** |  |
| Endotoxin assay | ≤ 0.5 EU/mL |
| Bovine serum albumin residuals | <50 ng/mL |

^a^The “Pharmacopoeia of the People’s Republic of China, Edition 2015, Volume III’’ was used as a reference for the testing methods.

**Supplementary Fig. S1**


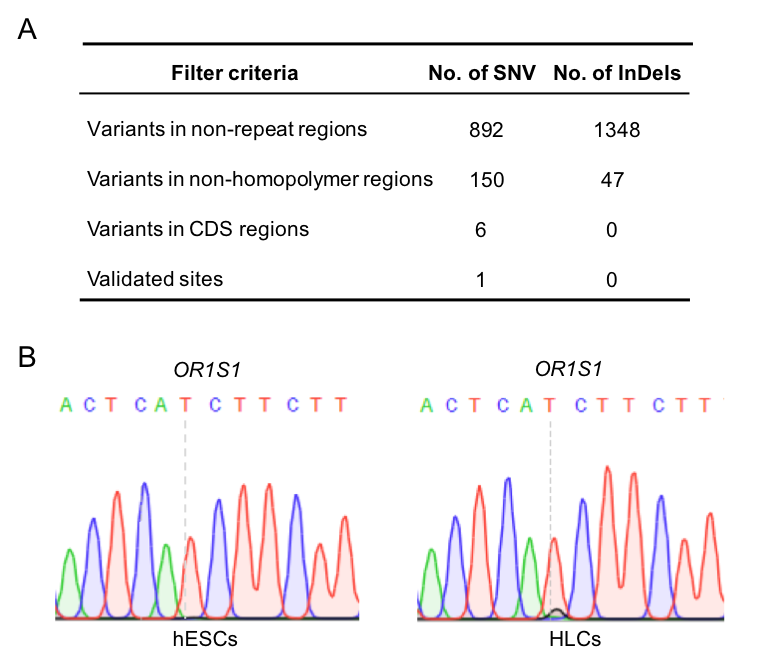


**Fig. S1** **The detection of single nucleotide variants (SNVs) and insertions and deletions (InDels) in HLCs through whole genome sequencing analysis.**

**a** The variants in non-repeat regions means the calling variants by GATK between HLCs and hESCs which located in repeat regions annotated by RepeatMasker (db20140131) were removed. And also the variants in non-homopolymer regions means the sites located within long base homopolymer (≥5bp) were omitted. **b** One sites could be validated by Sanger sequencing with about 10% mutation ratio which located in OR1S1 genes leading to amino acid changes in HLCs.

**Supplementary Fig. S2**


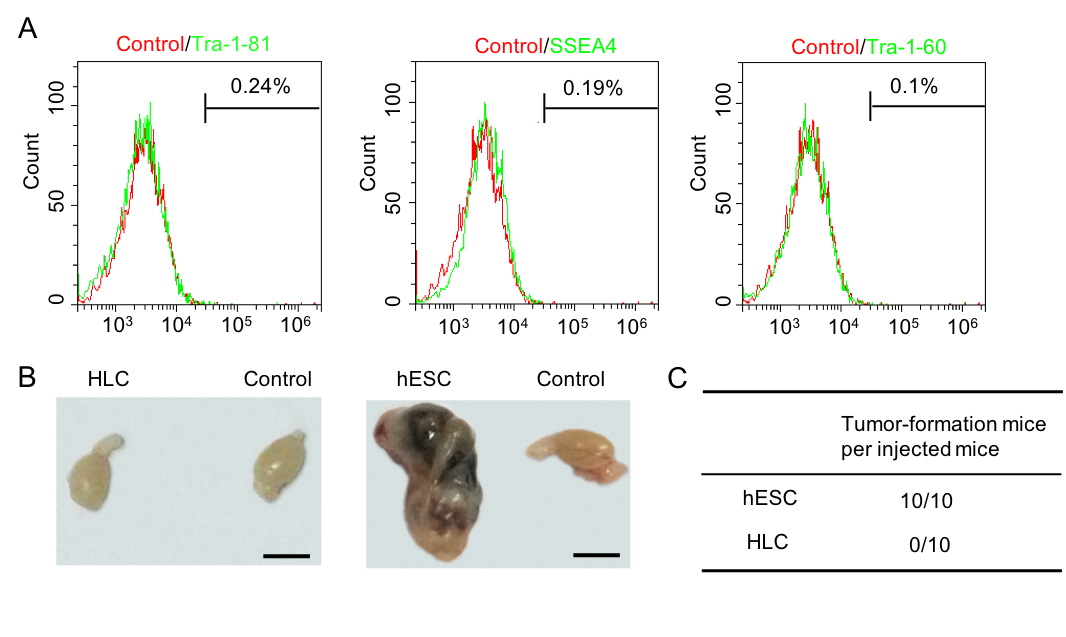


**Fig. S2 hESCs were not present among HLCs.**

**a** The expression levels of pluripotency genes in HLCs were determined by flow cytometry on day 19. Isotype control antibodies were used as controls. **b, c** HLCs (1×10^6^, n=10) appeared to be nontumorigenic after transplantation in SCID mouse testes compared with hESCs (1×10^6^, n=10) 2 months after transplantation. The scale bar represents 0.5 cm.

**Supplementary Fig. S3**


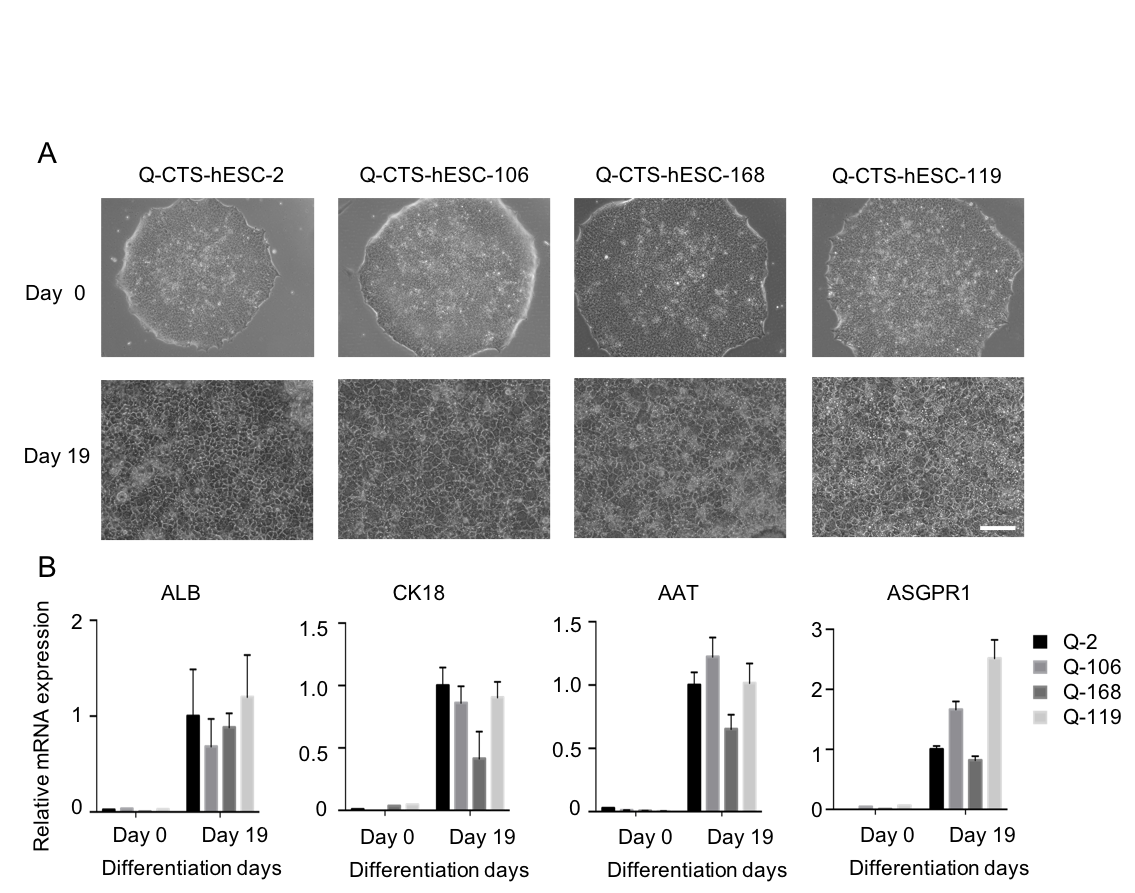


**Fig. S3 Differentiation of hepatocytes f****rom four human embryonic stem cell lines.**

**a** Representative morphology of cells on Days 0 and 19 from three other human embryonic stem cell lines (Q-CTS-hESC-106, Q-CTS-hESC-168 and Q-CTS-hESC-119), which were established similarly to the Q-CTS-hESC-2 cell line. **b** Expression levels of human hepatocyte-specific genes ALB, AAT, CK18 and ASGPR1 in HLCs from four human embryonic stem cell lines. The values were normalized to the total expression level of GAPDH. Data are represented as the mean ± SD, n=3. The scale bar represents 200 µm.

**Supplementary Fig. S4**


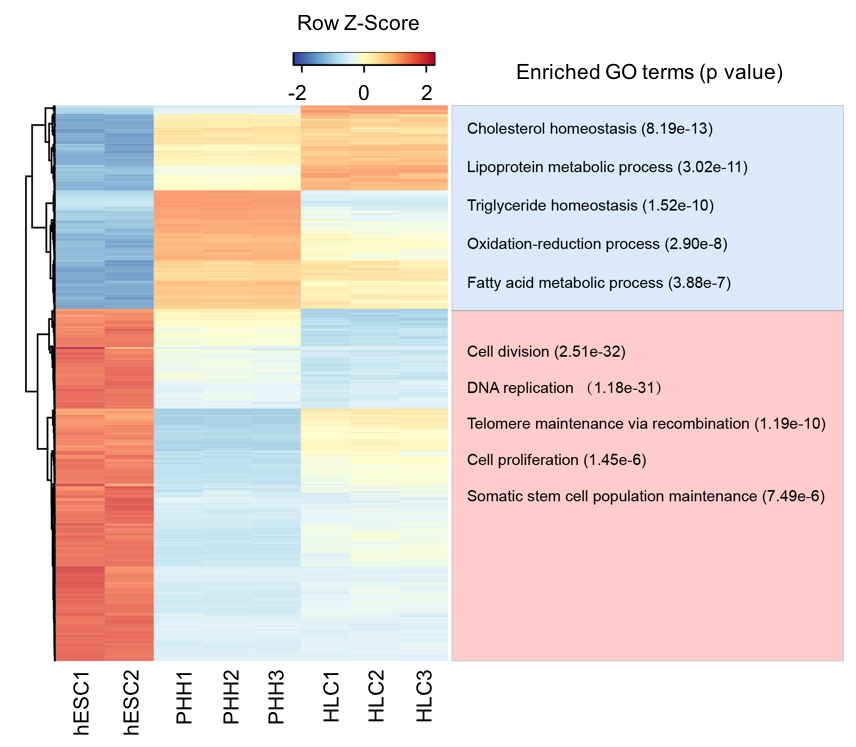


**Fig. S4** **The differentially expressed genes among hESCs and PHHs, HLCs.** In total, 2758 differentially expressed genes were found in hESCs compared to PHHs and HLCs. The enriched Gene Ontology (GO) terms and corresponding p values were marked.

**Supplementary Fig. S5**


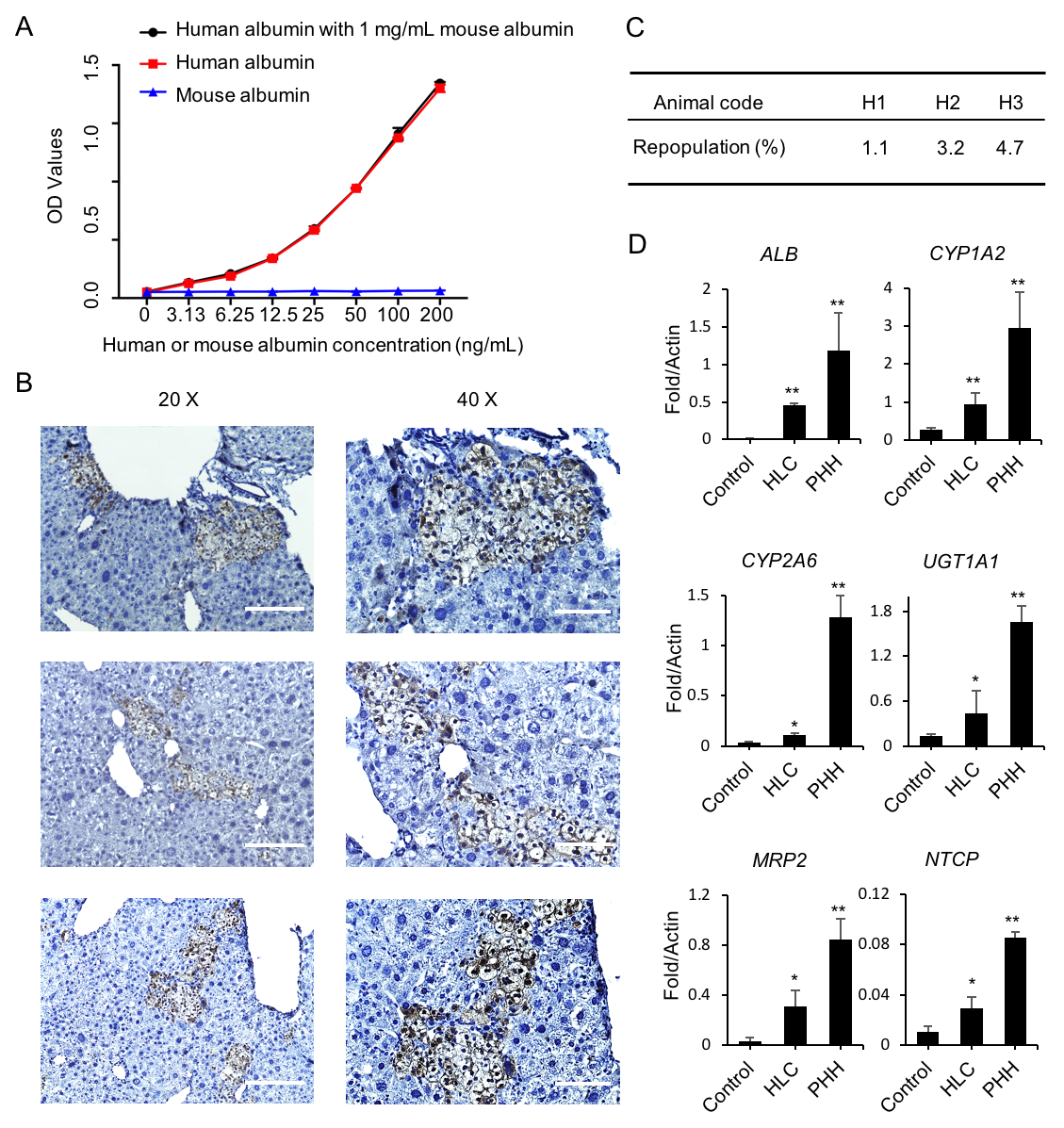


**Fig. S5 Repopulation of tet-uPA Rag2^-/-^ Il2rg^-/-^ mouse livers with HLCs.**

**a** The OD values of different concentrations of human serum albumin, mouse serum albumin and human serum albumin containing 1 mg/mL mouse serum albumin were determined. **b** The integration of HLCs in URG mouse livers was determined by immunostaining for human CK18 in serial sections. **c** Statistics of HLC repopulation in the livers of URG mice. **d** Expression levels of the human hepatocyte-specific gene *ALB* and metabolic genes, phase I enzymes *CYP1A2* and *CYP2A6*, phase II enzyme *UGT1A1*, and transporters *MRP2* and *NTCP* in the liver tissues from HLC- and PHH-transplanted URG mice (HLC, n=5; PHH, n=5) and PBS-injected URG mice (control, n=4). The values were normalized to total (mouse plus human) levels of *ACTIN*. For 20× magnification, the scale bar represents 200 µm; and for 40× magnification, the scale bar represents 100 µm. * p < 0.05, ** p < 0.01 compared to control; data are represented as the mean ± SD, n=3.

**Supplementary Table S3** Primer sequences used in this study.

| Gene name | Forward (5^’^-3’) | Reverse (5’-3’) |
| --- | --- | --- |
| *BRACHYURY* | TGCTTCCCTGAGACCCAGTT | GATCACTTCTTTCCTTTGCATCAAG |
| *MIXL1* | GGTACCCCGACATCCACTTG | TAATCTCCGGCCTAGCCAAA |
| *MESP2* | AGCTTGGGTGCCTCCTTATT | TGCTTCCCTGAAAGACATCA |
| *FOXA2* | GGAGCAGCTACTATGCAGAGC | CGTGTTCATGCCGTTCATCC |
| *SOX17* | CCAGAATCCAGACCTGCACAA | CTCTGCCTCCTCCACGAA |
| *Hand1* | GTGCGTCCTTTAATCCTCTTC | GTGAGAGCAAGCGGAAAAG |
| *HNF4α* | CTGCTCGGAGCCACCAAGAGATCCATG | ATCATCTGCCAGGTGATGCTCTGCA |
| *AFP* | AGAACCTGTCACAAGCTGTG | GACAGCAAGCTGAGGATGTC |
| *ALB* | CCTTTGGCACAATGAAGTGGGTAACC | CAGCAGTCAGCCATTTCACCATAGG |
| *AAT* | TATGATGAAGCGTTTAGGC | CAGTAATGGACAGTTTGGGT |
| *ASGPR1* | GAGAGAGACGTTCAGCAACTTC | GGGACTCTAGCGACTTCATCTT |
| *CK18* | TCGCAAATACTGTGGACAATGC | GCAGTCGTGTGATATTGGTGT |
| *SOX9* | AGCGAACGCACATCAAGAC | CTGTAGGCGATCTGTTGGGG |
| *CYP1A1* | TCGGCCACGGAGTTTCTTC | GGTCAGCATGTGCCCAATCA |
| *CYP1A2* | CTTCGCTACCTGCCTAACCC | GACTGTGTCAAATCCTGCTCC |
| *CYP2A6* | CAGCACTTCCTGAATGAG | AGGTGACTGGGAGGACTTGAGGC |
| *CYP2C8* | CATTACTGACTTCCGTGCTACAT | CTCCTGCACAAATTCGTTTTCC |
| *CYP2C9* | GCCTGAAACCCATAGTGGTG | GGGGCTGCTCAAAATCTTGATG |
| *CYP3A4* | TTCAGCAAGAAGAACAAGGACAA | GGTTGAAGAAGTCCTCCTAAGC |
| *FxR* | AACCATACTCGCAATACAGCAA | ACAGCTCATCCCCTTTGATCC |
| *AHR* | ACATCACCTACGCCAGTCG | CGCTTGGAAGGATTTGACTTGA |
| *CAR* | GTGCTCCTGTGCGGAGTAG | ATGGCAGATAGGCAGTTTCCC |
| *OATP1B3* | TGGAGCAACAGTACGGTCAG | TGCTTTCGCAGATTAGAGGGAA |
| *UGT1A1* | CATGCTGGGAAGATACTGTTGAT | GCCCGAGACTAACAAAAGACTCT |
| *UGT2B15* | CCAACCAATGAAGCCCCTG | GTTGTGAGCTGCGACTCGAA |
| *MGST1* | ATGACAGAGTAGAACGTGTACGC | TACAGGAGGCCAATTCCAAGA |
| *NNMT* | ATATTCTGCCTAGACGGTGTGA | TCAGTGACGACGATCTCCTTAAA |
| *NTCP* | AAGGACAAGGTGCCCTATAAAGG | TCAGTGACGACGATCTCCTTAAA |
| *MRP6* | AAGGAGGTACTAGGTGGGCTT | CCAGTAGGACCCTTCGAGC |
| *MRP2* | TCTCTCGATACTCTGTGGCAC | CTGGAATCCGTAGGAGATGAAGA |
| *GAPDH* | CTCTGCTCCTCCTGTTCGAC | CGACCAAATCCGTTGACTCC |
| *hALU* | CATGGTGAAACCCCGTCTCTA | GCCTCAGCCTCCCGAGTAG |
| *ACTIN** | TTCAACACCCCAGCCATG | CCTCGTAGATGGGCACAGT |

* Shared by human and mouse Actin.
